# Microstructure and Corrosion Behavior of Composite Coating on Pure Mg Acquired by Sliding Friction Treatment and Micro-Arc Oxidation

**DOI:** 10.3390/ma11071232

**Published:** 2018-07-18

**Authors:** Huihui Cao, Wangtu Huo, Shufang Ma, Yusheng Zhang, Lian Zhou

**Affiliations:** 1 College of Material Science and Engineering, Taiyuan University of Technology, Taiyuan 030024, China; caohuihui_92@163.com; 2 Northwest Institute for Nonferrous Metal Research, Xi’an 710016, China; zhoul@c-nin.com; 3 Department of Material Science and Engineering, Shaanxi University of Science & Technology, Xi’an 710021, China; mashufang@sust.edu.cn

**Keywords:** pure Mg, corrosion, sliding friction treatment, fine grain, micro-arc oxidation

## Abstract

For the purpose of detecting the influence of grain structure of a Mg matrix on the microstructure and corrosion resistance of micro-arc oxidation (MAO) coating, prior to MAO processing, sliding friction treatment (SFT) was adopted to generate a fine-grained (FG) layer on coarse-grained (CG) pure Mg surface. It showed that the FG layer had superior corrosion resistance, as compared to the CG matrix, owing to the grain refinement; furthermore, it successfully survived after MAO treatment. Thus, an excellent FG-MAO coating was gained by combining SFT and MAO. The surface morphology and element composition of FG-MAO and CG-MAO samples did not show significant changes. However, the FG layer favorably facilitated the formation of an excellent MAO coating, which possessed a superior bonding property and greater thickness. Consequently, the modified FG-MAO sample possessed enhanced corrosion resistance, since a lower hydrogen evolution rate, a larger impedance modulus and a lower corrosion current were observed on the FG-MAO sample.

## 1. Introduction

Commercial pure Mg and its alloys show enormous application potential in automotive, aviation and electric component fields, because of such factors as low density and excellent machinability [1,2,3]. They are also current prospective implant materials in orthopedics because of favorable biocompatibility, and a similar elastic modulus to that of human bone tissue [4,5,6]. However, poor resistance to corrosion has become a critical factor that severely restricts any further application.

For decades, numerous surface coating methods have been explored on Mg to enhance the anti-corrosion property [7,8,9,10,11,12]. In these methods, micro-arc oxidation (MAO) is a promising technique that could generate a protective ceramic coating in a suitable electrolyte [10,12,13,14,15]. Unfortunately, some disadvantages exist in current MAO coatings, including high porosity and relatively poor adhesive strength, which restricts wider application. Traditional methods for improving MAO coatings mainly include adjusting MAO operating parameters (e.g., applied voltage, oxidation time, etc.), altering the concentration and composition of the electrolyte, as well as sealing the holes through post-treatment [2,14,16,17,18]. Actually, the microstructure of the Mg matrix is extremely important for improving the quality of MAO samples. Many researchers confirm that increasing the defects (e.g., grain boundary, vacancy and dislocation) density of the substrate helps to accelerate coating growth rate [10,19,20], and the structure of MAO coatings is substrate grains dependent. It has been recently confirmed in Al [21] and Ti [13] alloys that the MAO coatings formed above fine-grained substrate possessed excellent corrosion resistance, as the extensive structural defects existing in the fine-grained substrate are capable of increasing the preferable nucleation sites for MAO coating and thus promoting the formation of superior coating with higher homogeneity and compactness. Therefore, it could also be expected that a modified MAO coating with higher protective function would be acquired for the above Mg-based materials with refined grains.

Severe plastic deformation (SPD) is a common and efficient technology to refine grains in various metals. Formerly, we studied an alternative SPD method (sliding friction treatment (SFT) [22,23]) which obtains refined grains on Ti [24], Al [25,26], Cu [23], Ta [27] and Mg [28] sheets. Therefore, we took SFT as the pretreatment process for pure Mg, prior to the MAO treatment, in order to fabricate the special composite coatings composed of the fine-grained (FG) layer above the coarse-grained (CG) matrix and the surface MAO ceramic coating. This work mainly studied the impact of the FG structure on the microstructure and degradation resistance of the aforementioned MAO coatings, and the corresponding mechanism was analyzed as well.

## 2. Materials and Methods

### 2.1. Materials and SFT Processing

A 200 × 200 × 5 mm^3^ commercially coarse-grained (CG) pure Mg (0.01% Al-0.01% Mn-0.0097% Fe-0.01% Si-0.0054% Zn-0.0001% Cu-0.0016% Ni) plate annealed at 450 °C for 30 h was used for SFT processing. Prior to SFT, the surface of the sheet was abraded by 500, 1000 and 1200# SiC sandpapers, ultrasonically degreased in acetone and ethyl alcohol for 5 min respectively, then rinsed with deionized water and quickly dried in the air. The details of the SFT set-up and specific processing parameters of SFT are presented in [28].

### 2.2. MAO Coating Preparation

The protective coating was manufactured by MAO process on the CG and SFT Mg samples. For MAO treatment, samples with a dimension of 10 × 10 × 5 mm^3^ were sandpapered down to 5000#, rinsed with ethyl alcohol and deionized water. During the MAO procedure, a pure Mg sample served as the anode and a stainless steel electric tank acted as the cathode. The electrolyte was composed of 10 g/L Na_2_SiO_3_·9H_2_O, 1 g/L KOH and 8 g/L KF·2H_2_O in deionized water. The temperature of the electrolyte stayed at room temperature through use of a stirring and cooling device during the entire procedure. The voltage and the frequency were set at 500 V and 100 Hz. The oxidation time and duty cycle were 2 min and 20%, respectively. The sample was washed with deionized water and dried in the air after MAO. MAO coatings that formed on the CG and FG surfaces were defined as CG-MAO and FG-MAO, respectively.

### 2.3. Characterization of Microstructure and Morphology

The microstructures of the CG and SFT sample were observed with optical microscopy (OM) and scanning electron microscope (SEM, Gemini 500, (ZEISS, Oberkochen, Germany)), respectively. The phases and element composition of substrates and coatings were analyzed by X-ray diffraction (XRD, D8 ADVANCE, (Bruker, Karlsruhe, Germany)) and X-ray photoelectron spectroscopy (XPS, ESCACAB 250XI, (Thermo Fisher, Waltham, MA, USA)). The morphology observation and elemental analysis of coatings were carried out by scanning electron microscope, equipped with energy dispersive spectroscopy. Before SEM test, all samples were treated with gold sputtering. 

The adhesion stabilities of CG-MAO and SFT-MAO coating were quantitatively evaluated using a WS-2005 scratch tester at room temperature. The load was linearly and continuously increased from 0 to 40 mN. The scratch length was 4 mm. A diamond with a diameter of 100 μm was drawn across the surface of the coatings at a speed of 0.04 mm/s. After scratch testing, the scratch morphology was examined with Zeiss Gemini 500 SEM.

### 2.4. Electrochemical Tests

The electrochemical workstation (IM6 Zahner-electrik Gmbh, (Zenniom, Kronach, Germany)) was adopted to study the corrosion behavior of samples by making use of potentiodynamic polarization (PDP) and electrochemical impedance spectroscopy (EIS) in simulated body fluid (SBF) electrolyte. During electrochemical tests, a saturated calomel electrode (SCE) and a platinum plate acted as reference electrode and assisting electrode, respectively. The samples serving as working electrode were embedded into epoxy resin with an area of 1 cm^2^ exposed to electrolyte. The SBF solution (pH: 7.42) was prepared using analytical reagent : 8.035 g/L NaCl, 0.355 g/L NaHCO_3_, 0.225 g/L KCl, 0.231 g/L K_2_HPO_4_·3H_2_O, 0.311 g/L MgCl_2_·6 H_2_O, 1.0 M HCl (39 mL), 0.292 g/L CaCl_2_, 0.072 g/L Na_2_SO_4_, 6.118 g/L Tris (hydroxymethyl ) aminomethane [11,29]. The temperature of SBF electrolyte was regulated at 37 °C. The EIS was tested from 10^5^ Hz to 0.1 Hz with AC voltage signal of 10 mV after immersion in SBF electrolyte for 30 min. The equivalent circuit was used to fit experimental EIS data by ZSimpWin software. The PDP curve was operated at a sweep rate of 0.01 Vs^−1^ from −2.0 to 0 V. The Tafel extrapolation method was employed to determine corrosion parameters. The corrosion rate can be obtained by Faraday’s law [30], CR = K(i_corr_/ρ)EW, where the unit of corrosion rate is mm/year, K is a constant and its value is 3.27 × 10^−3^ mm·g/(μA·cm·y), ρ and EW are the density (g/cm^3^) and equivalent weight of matrix, respectively.

### 2.5. Immersion Experiment

The degradation property was also estimated by immersion experiment in SBF electrolyte on the basis of ASTM-G31-72. All samples were embedded in resin with an area of 1 cm^2^ exposed to SBF electrolyte under a glass funnel connected with a burette. The hydrogen evolution volume (HEV) from the samples was measured from the water level of burette to evaluate corrosion rate. The hydrogen evolution rate v_H_ (HER) was obtained by the following Equation (1):

v_H_ = V/(st) (1)

where V and s are the hydrogen evolution volume (mL) and exposed area of samples (cm^2^), and t is immersion time (h). 

## 3. Results

### 3.1. Microstructure of Fine-Grained Modified Surface

**Figure 1:**
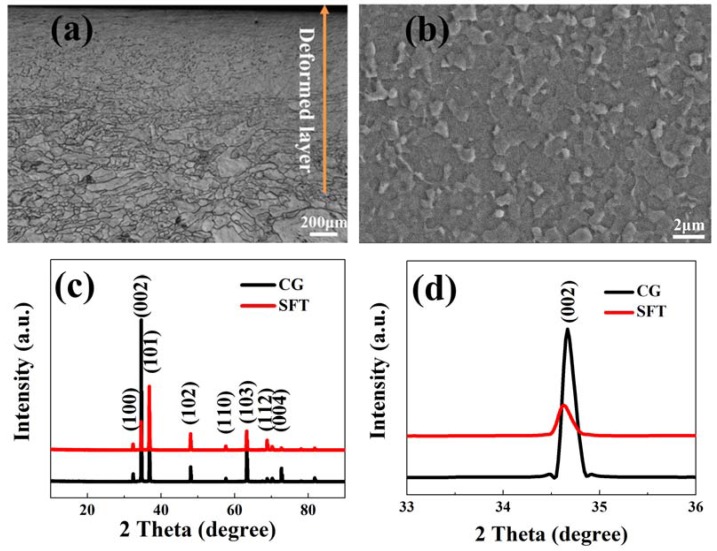
The cross-section (**a**) optical microscopy (OM) and (**b**) SEM picture of sliding friction treatment (SFT) sample; (**c**) XRD patterns of coarse-grained (CG) and SFT sample and (**d**) zoomed figure in the range of 33°–36°.

Figure 1a shows the optical microscope image of the cross-sectional SFT Mg sample. As can be seen, a 900 μm thick deformation layer is prepared on SFT sample surface where grain sizes are evidently refined. The average grain size of CG pure Mg is about 40 μm. Figure 1b presents an SEM image at the topmost part of the deformed layer. It is found that a great majority of grain sizes are between one and two microns. This FG structure fabricated on the CG Mg surface benefits from high strains in the SFT process [28,31].

The XRD graph of the CG and SFT sample is shown in Figure 1c. Mg is the main constituent for both the CG and SFT specimens. Further probing into the spectra of the diffraction (002) peak in the range of 33°–36° (Figure 1d), it is found that (002) peak becomes broadened, which is due to grain refinement induced by SFT treatment [32,33].

### 3.2. Microstructure Character of MAO Coating

**Figure 2:**
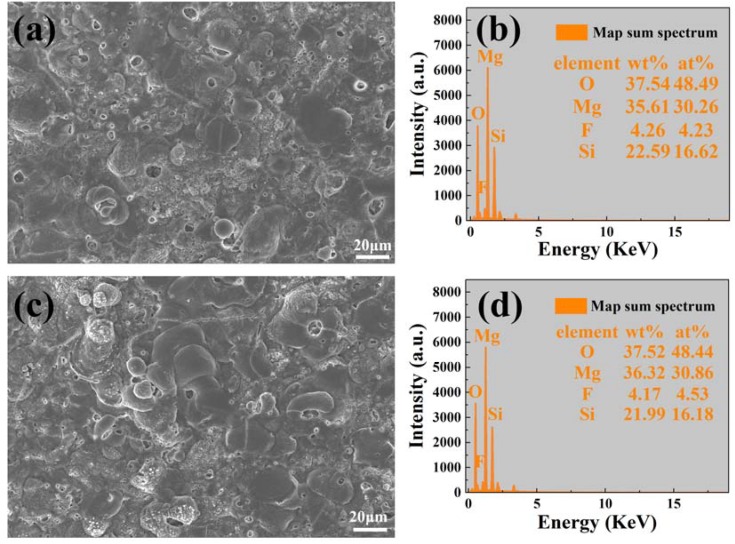
Surface morphology and EDS spectra of micro-arc oxidation (MAO) coatings: (**a**,**b**) coarse-grained micro-arc oxidation (CG-MAO); (**c**,**d**) fine-grained micro-arc oxidation (FG-MAO).

Figure 2 presents surface morphologies and corresponding EDS spectrums of FG-MAO and CG-MAO coatings. The typical micro-pore structure is formed on the surfaces of both coatings. The number and size of pores, along with the composition, exhibit no significant difference between the two coatings.

**Figure 3:**
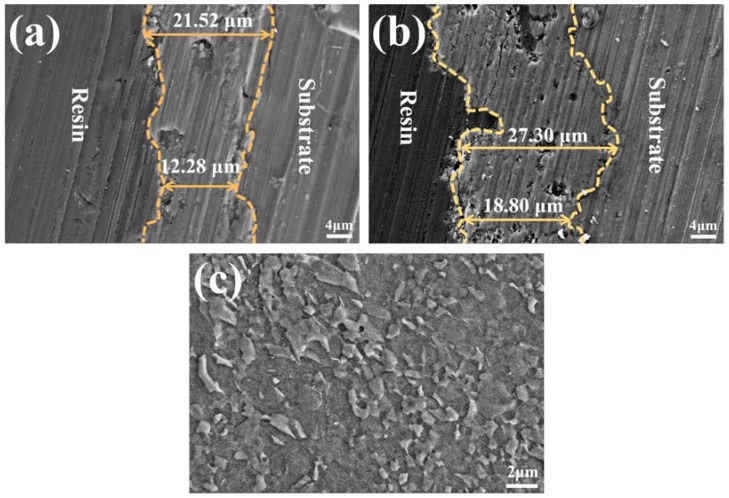
Cross-section SEM images of MAO coating: (**a**) CG-MAO and (**b**) FG-MAO; (**c**) SEM image underneath the coating in FG-MAO sample.

Figure 3a,b show cross-section images of two kinds of coatings. It is clear that the average thickness of the FG-MAO coating is 23.05 μm, which is evidently thicker than that of the MAO coating (16.62 μm). It has been proved that refined grains with enhanced density of high-energy grain boundaries and other defects can significantly accelerate the chemical reaction rate [12,13]. This finding means that the SFT pretreatment can accelerate the element diffusion rate and coating formation rate.

The microstructure underneath the coating in FG-MAO sample was also examined by SEM in Figure 3c. The average grain size of the substrate after MAO is 1~2 μm. It reveals that the grain size variation caused by MAO processing is almost negligible, consistent with the results of Refs. [12,34]. They confirm that local high temperature caused by micro-arc discharge did not cause grain growth of the FG matrix next to the MAO coating. This is also the first direct evidence that the FG layer successfully survived after MAO processing.

**Figure 4:**
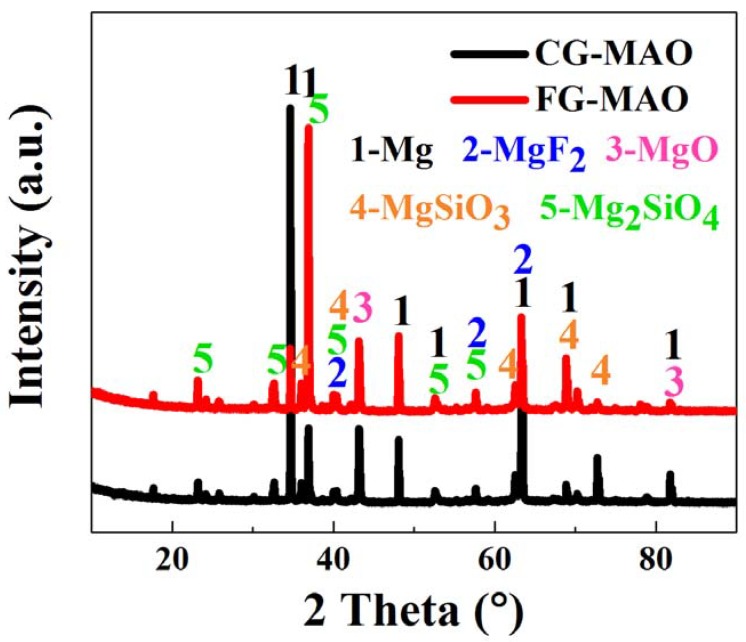
XRD graph of CG-MAO and FG-MAO sample prepared on pure Mg.

Figure 4 presents the X-ray diffraction pattern of both coatings. The phases of two kinds of coatings mainly consist of Mg, Mg_2_SiO_4_, MgF_2_, MgSiO_3_ and MgO. The element of Mg stems from Mg substrate. The appearance of Mg_2_SiO_4_, MgF_2_ and MgSiO_3_ compounds demonstrates that the Si, F and O of the solution have participated in the chemical reaction of the MAO process and have been incorporated into the coatings successfully. The existence of MgO may be ascribed to the high temperature induced by plasma discharge during the MAO process. The phase compositions of two coated samples in the XRD patterns show no obvious difference, which indicates that the SFT technology has little impact on the phase compositions of the coatings. 

**Figure 5:**
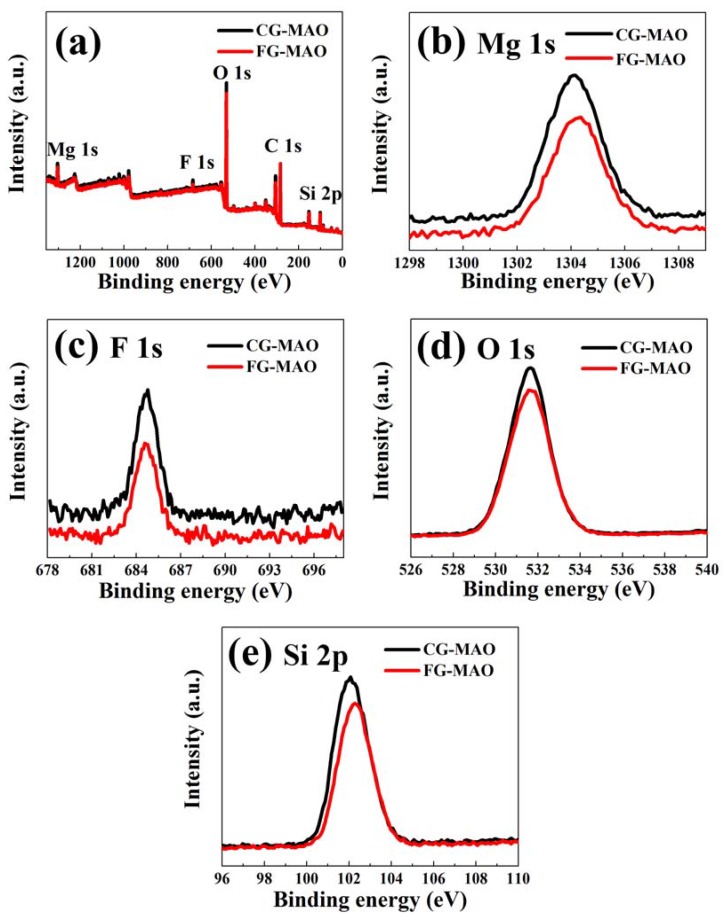
(**a**) X-ray photoelectron spectroscopy (XPS) survey spectra; (**b**) Mg 1s; (**c**) F 1s; (**d**) O 1s and (**e**) Si 2p high-resolution spectra for CG-MAO and FG-MAO samples.

Figure 5 presents the XPS and high-resolution spectra of CG-MAO and FG-MAO samples. The XPS survey spectra (Figure 5a) reveals that both coatings contain Mg, Si, O and F as the main elements. The binding energy value of these elements for both coatings is listed in Table 1. The binding energy of Mg1s peak is typical of magnesium in its oxidized form Mg^2+^ [35]. The binding energies of F1s peak at 685.17eV (CG-MAO coating) and 685.07 eV (FG-MAO coating) demonstrate the presence of MgF_2_ (685.4 eV) [36,37]. The Si2p peak at 102.07 eV (CG-MAO coating) and 102.27 eV (FG-MAO coating) are corresponding to MgSiO_3_ (102.3 eV) [38]. The XPS results are basically coincident with XRD.

**Figure 6:**
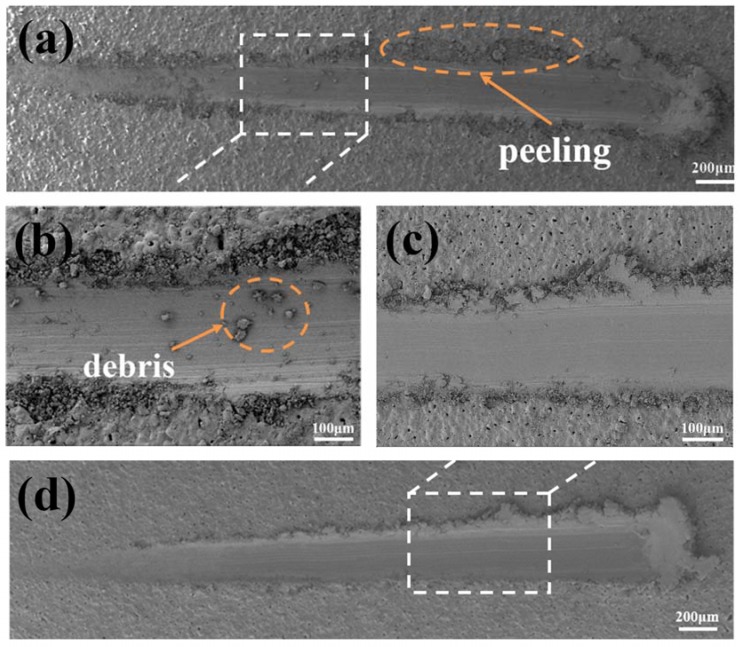
Scratch morphology of MAO samples: (**a**,**b**) CG-MAO and (**c**,**d**) FG-MAO.

Figure 6 shows the scratch appearance on both coatings. It is evident that serious delamination and peeling appear on the CG-MAO coating after the scratch test, as clearly visible debris can be observed inside the scratch groove. On the contrary, no evident peeling occurs on the FG-MAO coating surface along the entire scratch. Gu, et al. [34,39] have reported that the SPD-induced ultra-fine grained (UFG) layer on Ti and Al can offer tinier and denser discharge channels during MAO procedure and promote the formation of more compact coating. Similarly, we found that the SFT-induced FG layer on Mg can notably improve the adhesion performance between MAO coating and substrate. 

### 3.3. Corrosion Behavior

#### 3.3.1. Potentiodynamic Polarization

**Figure 7:**
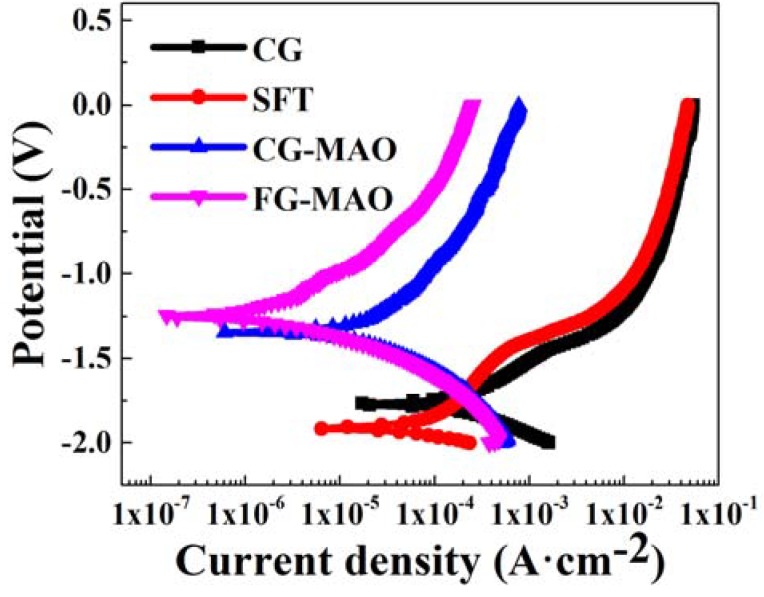
Potentiodynamic polarization curve of all samples immersed in simulated body fluid (SBF).

Figure 7 presents the PDP curve of CG, SFT, CG-MAO and FG-MAO samples exposed to SBF electrolyte. The corresponding corrosion parameters are summarized in Table 2. The degradation rate determined by Faraday’s law is listed in Table 3. The positive and negative potential relative to corrosion potential in curves represent the anode and cathode regions, corresponding to dissolution/passivation of the material and the hydrogen evolution, respectively [40,41]. The small peaks in curves for the CG and SFT sample indicate the transition between activation and passivation [40,42]. The corrosion tendency of materials can be predicted by corrosion potential (E_corr_), and the higher E_corr_ value represents lower corrosion susceptibility [1,30]. The E_corr_ (−1.255 ± 0.002 V) of FG-MAO coating is the highest in the midst of all samples, indicating FG-MAO coating is much more stable and has considerably less corrosion susceptibility. It is obvious that the I_corr_ (15.3 ± 3.2 μA/cm^2^) of the SFT sample is lower than that (31.6 ± 6.6 μA/cm^2^) of the CG sample. It is generally accepted that the corrosion current density is regarded as a vital index for estimating the kinetic of corrosion reactions [33]. Therefore, SFT pretreatment on the pure Mg sheet has a positive effect on corrosion resistance. This finding is also similar to the reports in Refs. [8,28] which highlighted enhanced corrosion resistance due to grain refinement. Besides, both CG and FG samples exhibit enhanced corrosion resistance after MAO, indicated by a more positive E_corr_ and lower I_corr_. I_corr_ value (0.35 ± 0.06 μA/cm^2^) of the FG-MAO sample, which is much lower compared to that of the CG-MAO sample. This result may be ascribed to the excellent adherence between the coating and substrate caused by refined grains produced by the SFT pretreatment. Among all samples, the I_corr_ of FG-MAO coating is two magnitudes lower than the FG sample and one order of magnitude lower than the CG-MAO coating. The corrosion rate of the FG-MAO sample is the lowest among all of the samples, which shows the comprehensive effect of grain refinement and the MAO process.

#### 3.3.2. Electrochemical Impedance Spectroscopy

**Figure 8:**
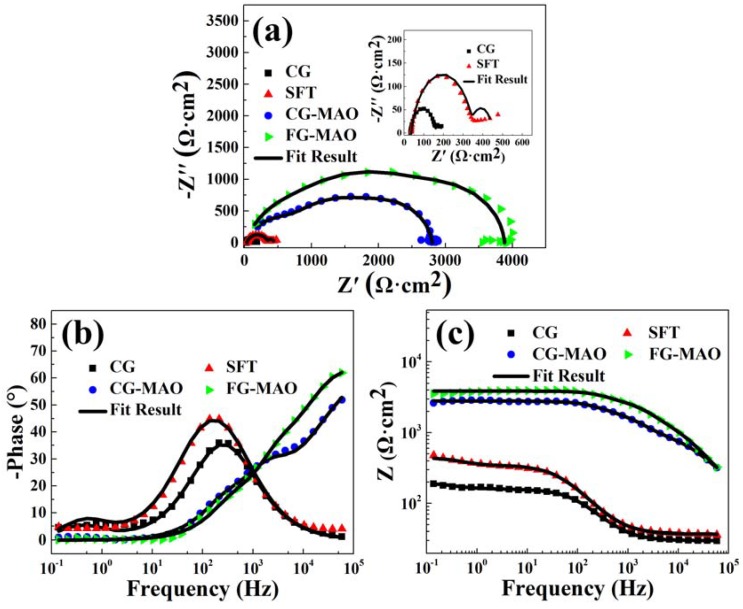
The electrochemical impedance spectroscopy (EIS) of all samples in SBF: (**a**) Nyquist plot; (**b**,**c**) Bode plots.

**Figure 9:**
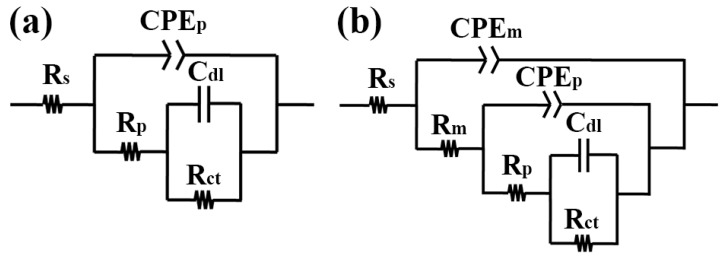
Equivalent circuits model for fitting EIS data of samples: (**a**) CG and SFT sample; (**b**) CG-MAO and FG-MAO coating.

The EIS measurements are carried out to further study the corrosion behavior of the samples [38]. Figure 8 displays Nyquist and Bode plots of all samples in SBF. It is evident that FG-MAO sample shows the biggest capacitive arc suggesting the optimal corrosion resisting property among all samples. Generally, the impedance module in regions of low frequency is considered as an important index in estimating the corrosion resistance [21,43]. The higher impedance modulus represents superior corrosion resisting property. The impedance value of the SFT sample is relatively higher than that of the CG sample, which indicates that SFT pretreatment leads to a mild enhancement of corrosion resisting property. It is apparent that the impedance module in regions of low frequency increased by one magnitude after coating, indicating that the corrosion resistance is enhanced significantly by MAO treatment. Meanwhile, the FG-MAO coating exhibits the biggest impedance value, demonstrating that combining the SFT and MAO technology provides more effective resistance to corrosion. On the basis of the EIS characteristics, the equivalent circuit models were employed for fitting impedance data. Figure 9 displays two kinds of equivalent circuits corresponding to uncoated and coated samples. R_s_ is the solution resistance. C_dl_ and R_ct_ represent electrochemical double layer capacitance and charge transfer resistance, respectively. CPE_p_ and R_p_ stand for the capacitance and resistance of naturally formed passive films. CPE_m_ and R_m_ correspond to the capacitance and resistance of the MAO coating. CPE is a kind of constant phase element, and its impedance values could be determined according to Equation (2):

Z_CPE_ = 1/[Q(jω)^n^] (2)

where j and ω correspond to imaginary unit and angular frequency, respectively. Q is a constant irrelative to frequency and n is exponential coefficient. Q equals capacitance and conductivity when n takes one and zero respectively. The EIS fitting parameters obtained from the equivalent circuits are listed in Table 4.

The R_ct_ of CG sample is the lowest of all samples, implying that SFT and MAO can improve the charge transfer resistance value. For the two samples subjected to the MAO treatment, R_m_ of the FG-MAO sample is nearly twice as much as the CG-MAO sample, indicating that the former shows superior corrosion resistance and that SFT pretreatment is beneficial for improving the quality of MAO coating.

#### 3.3.3. Immersion Tests

**Figure 10:**
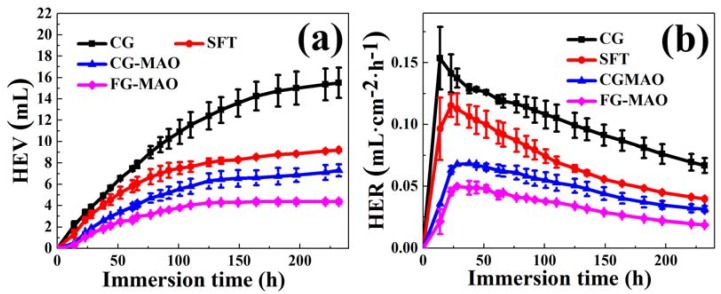
(**a**) Hydrogen evolution volume (HEV) and (**b**) hydrogen evolution rate (HER) curves of all samples immersed in SBF.

Figure 10 presents HEV and HER curves with increasing immersion time. It is clearly observed that the HEV and HER of samples in the SBF electrolyte for 230 h could be ranked as follows: the FG-MAO coating < the CG-MAO coating < the SFT sample < the CG sample. The result is consistent with electrochemical tests. This result indicates more effective corrosion resisting properties for the FG-MAO sample, over a long period of time. 

#### 3.3.4. Post-Corrosion Morphologies

**Figure 11:**
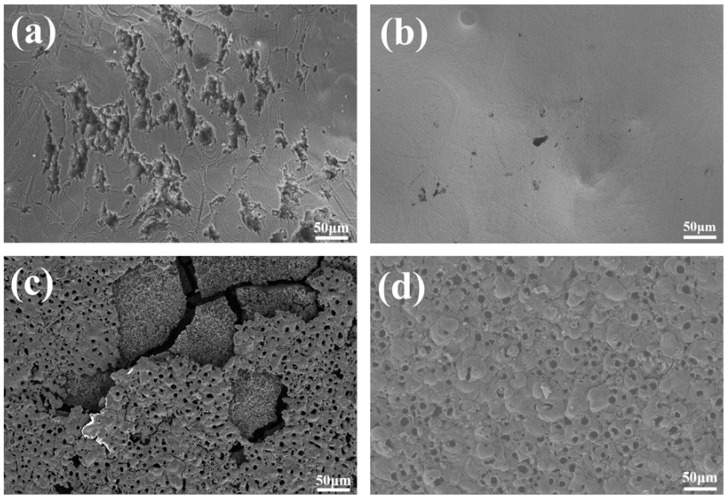
Post-corrosion morphologies of samples: (**a**) CG; (**b**) SFT; (**c**) CG-MAO; (**d**) FG-MAO.

Figure 11 shows post-corrosion SEM morphologies. The corrosion mechanisms of four kinds of samples are pitting corrosion. The formation of pitting results from the breakdown of passive films or MAO coatings on Mg or MAO sample surface under the effect of corrosive ions in the SBF electrolyte. As shown in Figure 11a,b, the CG sample presents severely localized corrosion morphology with huge corrosion pits on its surface, while the SFT sample exhibits a relatively smooth surface with only very slight pits. Also, the FG-MAO sample shows considerably lower levels of corrosion damage as compared to the CG-MAO coating. The comprehensive contribution from both SFT pretreatment and MAO process accounts for this favorable result.

## 4. Discussion

In this study, a composite coating composed of the fine-grained (FG) layer and MAO ceramic coating was acquired by SFT and MAO. After SFT pretreatment, a 900 μm thick deformation layer was prepared on pure Mg surface, in which the grain size was refined to the fine-grained level due to high strains in the SFT process. As shown in Figure 1, the phases of CG and SFT samples did not show any difference except for the broadened peak width caused by the effective grain refinement. The influence of SFT pretreatment on the corrosion resisting property of the Mg substrate was investigated as well. Based on electrochemical and immersion tests (Figure 7, Figure 8 and Figure 10), SFT Mg exhibits an obviously enhanced corrosion resistance, more so than that of the CG form, which is mainly ascribed to the grain refinement caused by SFT. FG with more grain boundaries helps to form a more dense protective oxide (MgO) film [12,21,44], which can usefully prevent Mg matrix against corrosive ions. The post-corrosion morphology of the FG sample with relatively smoother surface (Figure 11b) is the direct evidence to support this. Similar results are also discovered in lots of Mg treated by SPD techniques [10,21,45].

Furthermore, the influence of SFT-induced grain refinement on the microstructure and corrosion resisting property of MAO coating was studied as well. Results indicate SFT pretreatment helps to improve the quality and anti-corrosion property of MAO coating (Figure 6, Figure 7 and Figure 10).

It has been demonstrated that grain boundaries could serve as speedy diffusion channels for atoms and thus accelerating the chemical activity [31,34]. Thus, the driving power of MAO is improved for FG Mg from the point of view of thermodynamics. The results in Figure 3 and Figure 6 confirm that the SFT pretreatment promotes the formation the thicker coating with better bonding strength, which contribute to the significantly enhanced corrosion resistance and protect the Mg matrix from corrosion more effectively. Furthermore, this finding is similar to reports of Refs. [12,33,45]. Please note that the FG layer beneath the MAO coating should also have some effect in enhancing the overall degradation resistance of FG-MAO sample during the long period immersion. In case the external layer of MAO coating is penetrated by corrosive ions, a passive layer forms promptly in the damage area adjacent to FG layer, which could act as a partial compact layer of MAO coating and protect corrosive ions from penetrating into Mg matrix.

Results of electrochemical measurements clearly indicate that the FG-MAO sample possesses superior anti-corrosion property compared with CG-MAO sample. Specially, I_corr_ of FG-MAO coating is one magnitude lower. Meanwhile, R_m_ and R_ct_ of FG-MAO coating are evidently higher, offering more effective resistance to corrosion. The lower hydrogen evolution rate for FG-MAO coating reveals that FG-MAO sample can effectively prevent corrosive ions infiltrating into the interior of coating. Post-corrosion morphologies (Figure 11) in the SBF solution also show that the CG-MAO sample suffers from severe damage, while the FG-MAO coating still remains integrated structure. In general, the anti-degradation property of MAO coating is determined from its chemical composition, pore density, thickness and the adherent performance between the coating and matrix. The typical micro-pore structure (Figure 2) is formed on the surfaces of both coatings, and the number and size of pores exhibit no significant difference between two coatings. Base on the results of XRD (Figure 4) and XPS (Figure 5), the chemical composition and contents of the main elements for CG-MAO and FG-MAO coatings also show unclear difference. However, the FG-MAO coating exhibits higher thickness (Figure 3) and better bonding performance (Figure 6) as compared to the CG-MAO coating, which should account for the considerably enhanced corrosion resistance that was observed.

## 5. Conclusions

(1) A 900 μm thick deformation layer was manufactured on pure Mg surface by SFT technology. The grains existing in deformed layer were reduced to fine-grained level.
(2) MAO coatings were obtained successfully on CG and SFT samples. The surface morphologies and element compositions of both coatings do not exhibit visible difference. The FG-MAO sample has higher thickness and better interface bonding, which can be ascribed to the fast chemical reaction rate during MAO processing induced by FG structure.
(3) For all specimens, the FG-MAO sample shows the lowest corrosion rate, which means that SFT pretreatment is beneficial for improving the quality of MAO coating.

## Figures and Tables

**Table 1:** The binding energy of main elements of CG-MAO coating and FG-MAO coating.

Sample	Binding Energy (eV)			
	Mg	F	O	Si
CG-MAO	1304.07	685.17	531.67	102.07
FG-MAO	1304.37	685.07	531.87	102.27

**Table 2:** Main electrochemical parameters of all samples immersed in SBF solution.

Sample	E_corr_ (V vs. SCE)	i_corr_ (μA·cm^−2^)	β_c_ (mV·dec^−1^)	β_a_ (mV·dec^−1^)
CG	−1.772 ± 0.008	31.6 ± 6.6	53 ± 8	58 ± 12
SFT	−1.916 ± 0.012	15.3 ± 3.2	49 ± 12	69 ± 16
CG-MAO	−1.352 ± 0.004	2.75 ± 0.37	63 ± 10	75 ± 9
FG-MAO	−1.255 ± 0.002	0.35 ± 0.06	52 ± 7	99 ± 17

**Table 3:** The corrosion rate of samples calculated in accordance with Faraday’s law.

Sample	CG	SFT	CG-MAO	FG-MAO
CR (mm·year^−1^)	0.71 ± 0.14	0.34 ± 0.072	0.062 ± 0.007	0.008 ± 0.001

**Table 4:** Fitting parameters for Nyquist plot of the samples in SBF solution.

Sample	R_s_ (Ω·cm^2^)	R_m_ (Ω·cm^2^)	CPE_m_		R_p_ (Ω·cm^2^)	CPE_p_		R_ct_ (Ω·cm^2^)	C_dl_ (μF)
			Q (Ω^−1^·cm^−2^·s^n^)	n		Q (Ω^−1^·cm^−2^·s^n^)	n		
CG	2.98 ± 0.58	—	—	—	131 ± 24	(3.04 ± 0.38) × 10^−5^	0.82 ± 0.11	30.78 ± 4.22	139 ± 27
SFT	3.67 ± 0.86	—	—	—	315 ± 67	(2.48 ± 0.24) × 10^−5^	0.85 ± 0.14	93.52 ± 8.35	401 ± 36
CG-MAO	6.06 ± 1.23	1147 ± 102	(1.63 ± 0.15) × 10^−7^	0.76 ± 0.10	1108 ± 122	(1.43 ± 0.19) × 10^−7^	0.95 ± 0.18	543.91 ± 42.88	650 ± 43
FG-MAO	3.05 ± 0.81	1906 ± 135	(6.17 ± 0.72) × 10^−^^8^	0.84 ± 0.18	1212 ± 109	(5.23 ± 0.44) × 10^−^^8^	0.98 ± 0.10	736.95 ± 58.63	1300 ± 118

## References

[1] Chen J., Lan X., Wang C., Zhang Q. (2018). The Formation Mechanism and Corrosion Resistance of a Composite Phosphate Conversion Film on AM60 Alloy. Materials.

[2] Ma W., Liu Y., Wang W., Zhang Y. (2015). Effects of electrolyte component in simulated body fluid on the corrosion behavior and mechanical integrity of magnesium. Corros. Sci..

[3] Mordike B.L., Ebert T. (2001). Magnesium: Properties—Applications—Potential. Mater. Sci. Eng. A.

[4] Witte F., Kaese V., Haferkamp H., Switzer E., Meyer-Lindenberg A., Wirth C.J., Windhagen H. (2005). In vivo corrosion of four magnesium alloys and the associated bone response. Biomaterials.

[5] Xu L., Pan F., Yu G., Yang L., Zhang E., Yang K. (2009). In vitro and in vivo evaluation of the surface bioactivity of a calcium phosphate coated magnesium alloy. Biomaterials.

[6] Zheng Y.F., Gu X.N., Xi Y.L., Chai D.L. (2010). In vitro degradation and cytotoxicity of Mg/Ca composites produced by powder metallurgy. Acta Biomater..

[7] Abbas G., Liu Z., Skeldon P. (2005). Corrosion behaviour of laser-melted magnesium alloys. Appl. Surf. Sci..

[8] Chen L., Gu Y., Liu L., Liu S., Hou B., Liu Q., Ding H. (2015). Effect of ultrasonic cold forging technology as the pretreatment on the corrosion resistance of MAO Ca-P coating on AZ31B Mg alloy. J. Alloys Compd..

[9] Cheng W., Tian L., Ma S., Bai Y., Wang H. (2017). Influence of Equal Channel Angular Pressing Passes on the Microstructures and Tensile Properties of Mg-8Sn-6Zn-2Al Alloy. Materials.

[10] Gheytani M., Bagheri H.R., Masiha H.R., Aliofkhazraei M., Sabour Rouhaghdam A., Shahrabi T. (2014). Effect of SMAT preprocessing on MAO fabricated nanocomposite coating. Surf. Eng..

[11] Wang Y.M., Wang F.H., Xu M.J., Zhao B., Guo L.X., Ouyang J.H. (2009). Microstructure and corrosion behavior of coated AZ91 alloy by microarc oxidation for biomedical application. Appl. Surf. Sci..

[12] Xiong Y., Hu Q., Song R., Hu X. (2017). LSP/MAO composite bio-coating on AZ80 magnesium alloy for biomedical application. Mater. Sci. Eng. C.

[13] Gu Y., Chen L., Yue W., Chen P., Chen F., Ning C. (2016). Corrosion behavior and mechanism of MAO coated Ti6Al4V with a grain-fined surface layer. J. Alloys Compd..

[14] Mu W., Han Y. (2010). Study on Micro-Arc Oxidized Coatings on Magnesium in Three Different Electrolytes. Rare Met. Mater. Eng..

[15] Sankara Narayanan T.S.N., Park I.S., Lee M.H. (2014). Strategies to improve the corrosion resistance of microarc oxidation (MAO) coated magnesium alloys for degradable implants: Prospects and challenges. Prog. Mater. Sci..

[16] Duan H.P., Du K.Q., Yan C.W., Wang F. (2006). Electrochemical corrosion behavior of composite coatings of sealed MAO film on magnesium alloy AZ91D. Electrochim. Acta.

[17] Hussein R.O., Zhang P., Nie X., Xia Y., Northwood D.O. (2011). The effect of current mode and discharge type on the corrosion resistance of plasma electrolytic oxidation (PEO) coated magnesium alloy AJ62. Surf. Coat. Technol..

[18] Sabaghi Joni M., Fattah-alhosseini A. (2016). Effect of KOH concentration on the electrochemical behavior of coatings formed by pulsed DC micro-arc oxidation (MAO) on AZ31B Mg alloy. J. Alloys Compd..

[19] Youssef K.M.S., Koch C.C., Fedkiw P.S. (2004). Improved corrosion behavior of nanocrystalline zinc produced by pulse-current electrodeposition. Corros. Sci..

[20] Zhang X.Y., Shi M.H., Li C., Liu N.F., Wei Y.M. (2007). The influence of grain size on the corrosion resistance of nanocrystalline zirconium. Mater. Sci. Eng. A.

[21] Wen L., Wang Y., Zhou Y., Guo L., Ouyang J.H. (2011). Microstructure and corrosion resistance of modified 2024 Al alloy using surface mechanical attrition treatment combined with microarc oxidation process. Corros. Sci..

[22] Zhang Y.S., Niu H.Z., Zhang L.C., Bai X.F., Zhang X.M., Zhang P.X. (2014). Grain coarsening behavior in a nanocrystalline copper subjected to sliding friction. Mater. Lett..

[23] Zhang Y.S., Zhang P.X., Niu H.Z., Chen C., Wang G., Xiao D.H., Chen X.H., Yu Z.T., Yuan S.B., Bai X.F. (2014). Surface nanocrystallization of Cu and Ta by sliding friction. Mater. Sci. Eng. A.

[24] Lu J.W., Zhang Y.K., Huo W.T., Zhang W., Zhao Y.Q., Zhang Y.S. (2018). Electrochemical corrosion characteristics and biocompatibility of nanostructured titanium for implants. Appl. Surf. Sci..

[25] Chen Y.X., Yang Y.Q., Feng Z.Q., Huang B., Luo X., Zhang W. (2017). The depth-dependent gradient deformation bands in a sliding friction treated Al-Zn-Mg-Cu alloy. Mater. Charact..

[26] Chen Y.X., Yang Y.Q., Feng Z.Q., Zhao G.M., Huang B., Luo X., Zhang Y.S., Zhang W. (2017). Microstructure, microtexture and precipitation in the ultrafine-grained surface layer of an Al-Zn-Mg-Cu alloy processed by sliding friction treatment. Mater. Charact..

[27] Zhang Y.S., Wei Q.M., Niu H.Z., Li Y.S., Chen C., Yu Z.T., Bai X.F., Zhang P.X. (2014). Formation of nanocrystalline structure in tantalum by sliding friction treatment. Int. J. Refract. Met. Hard Mater..

[28] Huo W.T., Zhang W., Lu J.W., Zhang Y.S. (2017). Simultaneously enhanced strength and corrosion resistance of Mg–3Al–1Zn alloy sheets with nano-grained surface layer produced by sliding friction treatment. J. Alloys Compd..

[29] Kokubo T., Takadama H. (2006). How useful is SBF in predicting in vivo bone bioactivity?. Biomaterials.

[30] Ezhilselvi V., Nithin J., Balaraju J.N., Subramanian S. (2016). The influence of current density on the morphology and corrosion properties of MAO coatings on AZ31B magnesium alloy. Surf. Coat. Technol..

[31] Alsaran A., Purcek G., Hacisalihoglu I., Vangolu Y., Bayrak Ö., Karaman I., Celik A. (2011). Hydroxyapatite production on ultrafine-grained pure titanium by micro-arc oxidation and hydrothermal treatment. Surf. Coat. Technol..

[32] Liao Y., Cheng G.J. (2013). Controlled precipitation by thermal engineered laser shock peening and its effect on dislocation pinning: Multiscale dislocation dynamics simulation and experiments. Acta Mater..

[33] Zhang Y., You J., Lu J., Cui C., Jiang Y., Ren X. (2010). Effects of laser shock processing on stress corrosion cracking susceptibility of AZ31B magnesium alloy. Surf. Coat. Technol..

[34] Yao Z.Q., Ivanisenko Y., Diemant T., Caron A., Chuvilin A., Jiang J.Z., Valiev R.Z., Qi M., Fecht H.J. (2010). Synthesis and properties of hydroxyapatite-containing porous titania coating on ultrafine-grained titanium by micro-arc oxidation. Acta Biomater..

[35] Zhang C.L., Zhang F., Song L., Zeng R.C., Li S.Q., Han E.H. (2017). Corrosion resistance of a superhydrophobic surface on micro-arc oxidation coated Mg-Li-Ca alloy. J. Alloys Compd..

[36] Mao L., Shen L., Chen J., Wu Y., Kwak M., Lu Y., Xue Q., Pei J., Zhang L., Yuan G., Fan R., Ge J., Ding W. (2015). Enhanced bioactivity of Mg-Nd-Zn-Zr alloy achieved with nanoscale MgF_2_ surface for vascular stent application. ACS Appl. Mater. Inter..

[37] Li Z., Shizhao S., Chen M., Fahlman B.D., Debao L., Bi H. (2017). In vitro and in vivo corrosion, mechanical properties and biocompatibility evaluation of MgF_2_-coated Mg-Zn-Zr alloy as cancellous screws. Mater. Sci. Eng. C.

[38] Pan Y., Chen C., Wang D., Huang D. (2014). Dissolution and precipitation behaviors of silicon-containing ceramic coating on Mg-Zn-Ca alloy in simulated body fluid. Colloids and Surf. B Biointerfaces.

[39] Gu Y., Ma H., Yue W., Tian B., Chen L., Mao D. (2016). Microstructure and corrosion model of MAO coating on nano grained AA2024 pretreated by ultrasonic cold forging technology. J. Alloys Compd..

[40] Wang R., He X., Gao Y., Zhang X., Yao X., Tang B. (2017). Antimicrobial property, cytocompatibility and corrosion resistance of Zn-doped ZrO_2_/TiO_2_ coatings on Ti6Al4V implants. Mater. Sci. Eng. C.

[41] Dou J., Gu G., Chen C. (2017). Effects of calcium salts on microstructure and corrosion behavior of micro-arc oxidation coatings on Mg-2Zn-1Ca-0.8 Mn alloy. Mater. Lett..

[42] Zeng R.C., Cui L.Y., Jiang K., Liu R., Zhao B.D., Zheng Y.F. (2016). In Vitro Corrosion and Cytocompatibility of a Microarc Oxidation Coating and Poly(l-lactic acid) Composite Coating on Mg-1Li-1Ca Alloy for Orthopedic Implants. ACS Appl. Mater. Interfaces.

[43] Wang L.Q., Zhou J.S., Liang J., Chen J.M. (2012). Microstructure and corrosion behavior of plasma electrolytic oxidation coated magnesium alloy pre-treated by laser surface melting. Surf. Coat. Technol..

[44] Afshari V., Dehghanian C. (2009). Effects of grain size on the electrochemical corrosion behaviour of electrodeposited nanocrystalline Fe coatings in alkaline solution. Corros. Sci..

[45] Jiang J., Zhou Q., Yu J., Ma A., Song D., Lu F., Zhang L., Yang D., Chen J. (2013). Comparative analysis for corrosion resistance of micro-arc oxidation coatings on coarse-grained and ultra-fine grained AZ91D Mg alloy. Surf. Coat. Technol..

